# Two-stage repair for DeBakey type II acute aortic dissection and distal aortic arch aneurysm in a nonagenarian patient

**DOI:** 10.1093/jscr/rjad648

**Published:** 2023-12-05

**Authors:** Kentaro Shirakura, Shingo Kunioka, Kazuki Miyatani, Nobuhiro Mochizuki, Hideki Isa, Yuki Setogawa, Fumitaka Suzuki, Ryo Okubo, Ryohei Ushioda, Aina Hirofuji, Masahiro Tsutsui, Natsuya Ishikawa, Hiroyuki Kamiya

**Affiliations:** Department of Cardiac Surgery, Asahikawa Medical University, Midorigaoka-higashi 2-1-1-1, Asahikawa, Hokkaido, 078-8510, Japan; Department of Cardiac Surgery, Asahikawa Medical University, Midorigaoka-higashi 2-1-1-1, Asahikawa, Hokkaido, 078-8510, Japan; Department of Cardiac Surgery, Asahikawa Medical University, Midorigaoka-higashi 2-1-1-1, Asahikawa, Hokkaido, 078-8510, Japan; Department of Cardiac Surgery, Asahikawa Medical University, Midorigaoka-higashi 2-1-1-1, Asahikawa, Hokkaido, 078-8510, Japan; Department of Cardiac Surgery, Asahikawa Medical University, Midorigaoka-higashi 2-1-1-1, Asahikawa, Hokkaido, 078-8510, Japan; Department of Cardiac Surgery, Asahikawa Medical University, Midorigaoka-higashi 2-1-1-1, Asahikawa, Hokkaido, 078-8510, Japan; Department of Cardiac Surgery, Asahikawa Medical University, Midorigaoka-higashi 2-1-1-1, Asahikawa, Hokkaido, 078-8510, Japan; Department of Cardiac Surgery, Asahikawa Medical University, Midorigaoka-higashi 2-1-1-1, Asahikawa, Hokkaido, 078-8510, Japan; Department of Cardiac Surgery, Asahikawa Medical University, Midorigaoka-higashi 2-1-1-1, Asahikawa, Hokkaido, 078-8510, Japan; Department of Cardiac Surgery, Asahikawa Medical University, Midorigaoka-higashi 2-1-1-1, Asahikawa, Hokkaido, 078-8510, Japan; Department of Cardiac Surgery, Asahikawa Medical University, Midorigaoka-higashi 2-1-1-1, Asahikawa, Hokkaido, 078-8510, Japan; Department of Cardiac Surgery, Asahikawa Medical University, Midorigaoka-higashi 2-1-1-1, Asahikawa, Hokkaido, 078-8510, Japan; Department of Cardiac Surgery, Asahikawa Medical University, Midorigaoka-higashi 2-1-1-1, Asahikawa, Hokkaido, 078-8510, Japan

**Keywords:** aortic dissection, thoracic artery aneurysm, TEVAR

## Abstract

Although total arch replacement would be performed in a patient with acute type A aortic dissection and concomitant aortic aneurysm in the distal aortic arch, total arch replacement may be too invasive in elderly patients with significant morbidities. A 92-year-old female with acute type II DeBakey aortic dissection and concomitant distal aortic arch aneurysm was successfully treated with hemi-arch replacement followed by thoracic endovascular aortic repair. Hybrid two-stage repair of DeBakey type II aortic dissection complicated by distal arch aneurysm using thoracic endovascular aortic repair after hemi-arch replacement may be effective.

## Introduction

Normally, total arch replacement is performed in patients with acute type A aortic dissection and a concomitant aortic aneurysm in the distal aortic arch. However, total arch replacement may be too invasive in older patients with significant morbidities due to prolonged aortic cross-clamp time, hypothermic circulatory arrest time, bleeding risk, and the risk of aspiration pneumonia resulting from left recurrent laryngeal nerve palsy. We report the case of a 92-year-old female with DeBakey type II acute aortic dissection and concomitant distal aortic arch aneurysm that was successfully treated with hemiarch replacement and subsequent thoracic endovascular aortic repair (TEVAR).

## Case report

**Figure 1:**
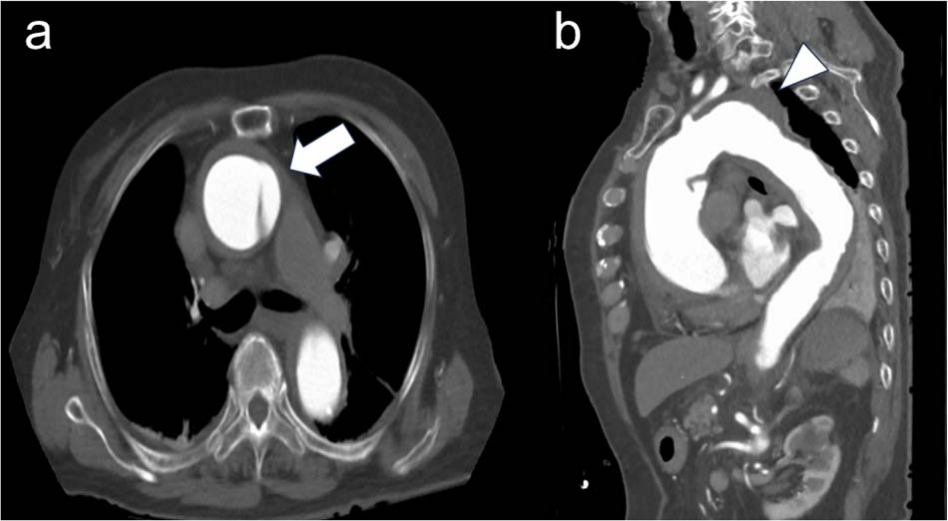
Preoperative computed tomography imaging. (a) There was a flap at the ascending aorta, a finding of acute aortic dissection of Stanford type A (white arrow). (b) A 54 mm large thoracic aortic aneurysm was found at the distal arch aorta (white arrow head).

A 92-year-old female presenting with acute chest pain was transferred to our institution because of a Stanford type A aortic dissection. Contrast-enhanced computed tomography (CT) revealed a distal aortic arch aneurysm measuring 54 mm in diameter (Fig. 1).

Her blood pressure was 180/120 mmHg and pulse were 80/min. We had initially planned for total aortic arch replacement using the frozen elephant trunk technique; however, considering the patient’s age, we decided to use hemiarch replacement for the ascending aorta in aortic dissection and the distal aneurysm was follow-up via another round of CT. This approach was performed using a median sternotomy. The arterial cannulation was performed using Seldinger’s technique on the ascending aorta and venous drainage for cardiopulmonary bypass (CPB) was performed via the superior vena cava and inferior vena cava. After cannulation was completed, CPB and systemic cooling were initiated. Systemic perfusion was stopped by retrograde cardioplesia when the core body temperature reached 28°C and retrograde cerebral perfusion was given through internal jugular vein. The ascending aorta was replaced with a 26 mm J graft (Japan Lifeline, Tokyo, Japan).

The patient had an uneventful postoperative course during her hospital stay and was discharged on postoperative Day 22. However, 2 days after discharge, the patient presented with back pain and chest discomfort. We observed no significant changes in the aneurysm on contrast-enhanced CT. We suspected impending rupture and decided to treat it with 1-debranching TEVAR to relieve symptoms and prevent rupture. Under general anaesthesia, a left common carotid artery–left subclavian artery bypass using a ringed 6-mm expanded polytetrafluoroethylene graft (PROPATEN®, WL Gore & Associates, Flagstaff, AZ) was established prior to TEVAR. The patient underwent TEVAR using an 38 × 217 mm endoprosthesis (Cook Zenith Alpha®, Cook Medical, Bloomington, IN, USA). The device was passed through the left femoral artery.

**Figure 2:**
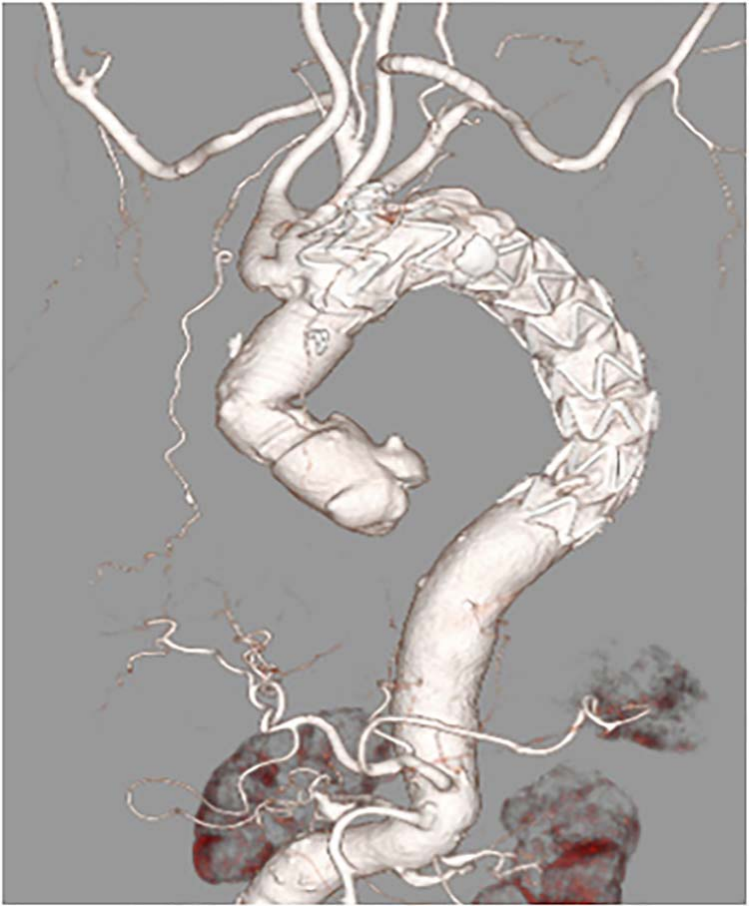
Postoperative enhanced-computed tomography findings. There were no problematic findings, although a slight type 4 endoleak was present.

After primary aortography was performed using a 5-F pigtail catheter, the landing zones were marked; the introducer was inserted; and the endoprosthesis was tracked and centred over the aneurysm under fluoroscopic guidance, deployed and subsequently ballooned in Zone 2. Angiography revealed a type 1 endoleak; therefore, inflation with a trilobe balloon was performed. Two 8 × 20 cm coils (Interlock™, Boston Scientific, Marlborough, Massachusetts) were introduced through the left subclavian artery into the aneurysms for embolization. The final angiogram showed a slight leak in the aneurysm; however, the blood flow was determined to be from the left subclavian artery, and the procedure was terminated. Although contrast-enhanced CT performed on postoperative Day 8 showed a type 4 endoleak (Fig. 2). The postoperative course was uneventful, and she was discharged on Day 15.

## Discussion

Despite recent developments in surgical techniques and perioperative management, the conventional distal arch repair for aortic aneurysms is invasive, particularly for older patients. TEVAR has been established as a less invasive alternative relative to the conventional open surgical repair for the management of descending thoracic aortic diseases. Here, we report our treatment strategy using TEVAR to efficiently prevent postoperative complications in an older patient.

Reportedly, 5%–20% cases of aortic dissection are associated with arteriosclerotic aneurysms [1]. In recent years, the incidence of such cases in older population is increasing considering the advancing societal age. Type A aortic dissection is associated with critical complications, and determining the type of surgical procedure required is difficult in some cases. In particular, when an aortic dissection overlaps with an aortic aneurysm, the risk of aortic aneurysm rupture in the postoperative period could be high if the aneurysm is left untreated. Although the median sternotomy approach enables peripheral anastomosis or the use of an elephant trunk, the risk of spinal cord ischaemia by covering the main vessels feeding the spinal cord in the case of open stent graft surgery during circulatory arrest is as high as 0%–24% while this number reduces to 0%–8.3% with TEVAR [2, 3]. In the present case, the aortic dissection lesion expanded only on ascending aorta and the distal aortic arch aneurysm remained enough landing to treat with TEVAR on zone 2. Therefore, peripheral anastomosis was performed at the end of the ascending aorta, which was in a relatively good condition for the initial surgery. Accordingly, the surgery could be performed without involving the aortic arch, which was markedly atherosclerotic. Furthermore, when combined with TEVAR, it is relatively minimally invasive, with a shorter time to complete surgery compared with TAR in the first stage. A study comparing the outcome of post TAR patients aged 80 years or more and those <80 years revealed that in the former group, operative death and 1-year mortality were 8.6% and 27.2%, respectively. Additionally, due to high rates of other complications, significantly fewer patients in the age group of 80 years or more were discharged [4]. If we focused on the results of nonagenarian patients, these numbers would be higher. Therefore, the surgical strategy should consider the extent of surgical invasiveness and long-term prognosis within the context of the patient’s life expectancy. To apply this method, sufficient proximal and distal landing zones of at least 2 cm are necessary for safe deployment and durable fixation using TEVAR. The stent–graft diameter should exceed the diameter of the landing zones by at least 10%–15% of the reference aortic diameter. Given the right conditions, such as those in this case, we could conduct a two-stage procedure that is less invasive and can be adopted even for super-elderly (e.g. 85 years and over) patients.
